# In this month’s Bulletin

**DOI:** 10.2471/BLT.17.000517

**Published:** 2017-05-01

In the editorial section, Mahmood F Bhutta (314) introduces a campaign to improve conditions for workers who manufacture surgical instruments, gloves and textiles. Grania Brigden et al. (315) announce a new funding mechanism for research and development of tuberculosis medicines.

In the news section, Tatum Anderson (318–319) reports on efforts to prevent deaths from prescription opioids in Canada and the United States of America. Fiona Fleck (320–321) interviews Svetlana Popova about her research on the global costs and consequences of maternal alcohol consumption during pregnancy.

## Bangladesh 

### Essential medicines for noncommunicable diseases

Sheikh Mohammed Shariful Islam et al. (382–384) explore ways to improve national drug policies.

## India

### Telemedicine and social franchising

Manoj Mohanan et al. (343–352) study service provision for the treatment of diarrhoea and pneumonia in children.

## Kenya

### Ensuring good outcomes

Miriam Rabkin et al. (353–361) evaluate nurses’ provision of HIV services. 

## Madagascar 

### Recording influenza cases in the tropics

Alain Rakotoarisoa et al. (375–381) describe a sentinel surveillance system. 

## Malawi

### Tobacco crops and sustainable development

Margarete C Kulik et al. (362–367) examine health, environmental and economic impacts of tobacco farming. 

## Sub-Saharan Africa

### Does everyone receive a net?

Cameron Taylor et al. (322–332) track inequities in ownership of mosquito nets. 

## Zambia

### Seeking care in rural areas

Karsten Lunze et al. (333–342) assess the quality of care provided to children presenting with fever.

## Global

### Preventing obesity and malnutrition

Hélène Delisle et al. (385–388) argue that more nutritionists are needed.

### Measuring quality of care

Johanna Hanefeld et al. (368–374) list problems and solutions for low- and middle-income countries.

